# Three microarray platforms: an analysis of their concordance in profiling gene expression

**DOI:** 10.1186/1471-2164-6-63

**Published:** 2005-05-05

**Authors:** David Petersen, GVR Chandramouli, Joel Geoghegan, Joanne Hilburn, Jonathon Paarlberg, Chang Hee Kim, David Munroe, Lisa Gangi, Jing Han, Raj Puri, Lou Staudt, John Weinstein, J Carl Barrett, Jeffrey Green, Ernest S Kawasaki

**Affiliations:** 1 Advanced Technology Center, Center for Cancer Research, National Cancer Institute, Gaithersburg, MD 20877 USA; 2 Laboratory of Molecular Technology, SAIC Frederick, Frederick, MD 21701 USA; 3 Center for Biologics Evaluations & Research, Food & Drug Administration, Bethesda, MD 20892 USA; 4 Center for Cancer Research, National Cancer Institute, Bethesda, MD 20892 USA

## Abstract

**Background:**

Microarrays for the analysis of gene expression are of three different types: short oligonucleotide (25–30 base), long oligonucleotide (50–80 base), and cDNA (highly variable in length). The short oligonucleotide and cDNA arrays have been the mainstay of expression analysis to date, but long oligonucleotide platforms are gaining in popularity and will probably replace cDNA arrays. As part of a validation study for the long oligonucleotide arrays, we compared and contrasted expression profiles from the three formats, testing RNA from six different cell lines against a universal reference standard.

**Results:**

The three platforms had 6430 genes in common. In general, correlation of gene expression levels across the platforms was good when defined by concordance in the direction of expression difference (upregulation or downregulation), scatter plot analysis, principal component analysis, cell line correlation or quantitative RT-PCR. The overall correlations (r values) between platforms were in the range 0.7 to 0.8, as determined by analysis of scatter plots. When concordance was measured for expression ratios significant at p-values of <0.05 and at expression threshold levels of 1.5 and 2-fold, the agreement among the platforms was very high, ranging from 93% to 100%.

**Conclusion:**

Our results indicate that the long oligonucleotide platform is highly suitable for expression analysis and compares favorably with the cDNA and short oligonucleotide varieties. All three platforms can give similar and reproducible results if the criterion is the direction of change in gene expression and minimal emphasis is placed on the magnitude of change.

## Background

Completion of the human genome sequence has made it possible to study expression of the entire complement of 20,000–30,000 genes in a single assay. The two most common array platforms are based on collections of cDNA clones [1] or short (25 base) oligonucleotides synthesized *in situ *by photolithographic methods (i.e., by Affymetrix, Inc.) [2]. Partly because they are easy to use, microarrays are the most extensively used technology for studying gene expression on a global scale [3,4]. Thousands of expression studies employ one or the other microarray platform, but comparison of results between platforms has been difficult because of inherent differences in the array technologies. The situation became more complex as investigators began using long oligonucleotide arrays for expression profiling [5-9].

Because long oligonucleotide arrays for expression profiling are relatively new, we wished to validate them in relation to the cDNA and short oligonucleotide platforms, both of which have been used extensively in our laboratories over a number of years. The three platforms were evaluated using RNAs isolated from six cell lines and tested against a universal reference RNA. Sufficient RNA was isolated in a single harvest to supply labeling template for all experiments, so variability of RNA isolation was not an issue. However, no attempt was made to eliminate other sources of variation such as differences between lots of fluorescent dye label, microarray batch, operator, etc. We conducted these experiments under "normal" laboratory conditions so that one would not need to go to extreme lengths to reproduce the results. In almost all cases, results from the three platforms correlated reasonably well with each other. The Pearson correlation coefficients (r) ranged from 0.7 to 0.8. Because of different labeling methods and analysis algorithms, comparison of the cDNA and long oligonucleotide platforms with the short oligonucleotide system was not as straightforward, but in general all of the platforms were in reasonable agreement.

## Results

This study was carried out to compare cDNA (Incyte), long oligonucleotide (Operon 70-mer), and short 25-mer (Affymetrix) array platforms, with the goal of qualifying the 70-mer arrays for general use at the National Cancer Institute. More specifically, we compared the Incyte Unigem2 set of human cDNAs (~9900 genes), the Operon human Version 2.0 set of long oligonucleotides (~21,329 genes), and Affymetrix HG-U133A arrays (~22,200 genes). RNA preparations from cell lines MCF10A, LNCaP, Jurkat, L428, SUDHL6, and OCI-Ly3 were used as probe templates, and the expression of each gene was compared directly with that of the same gene in the Human Universal Reference (HUR) RNA from Stratagene.

### Genes in common across platforms

**Figure 1:**
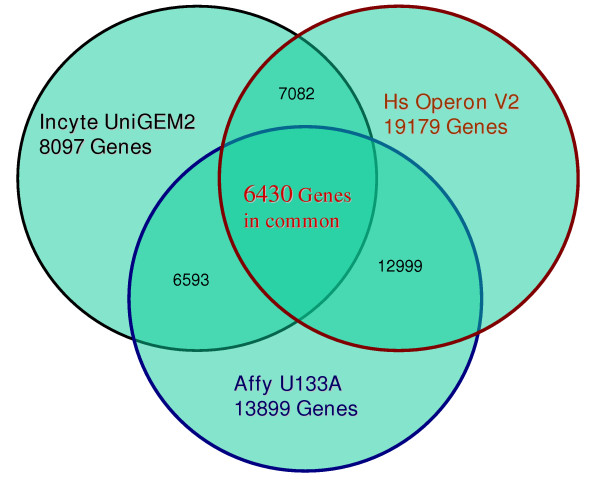
Venn diagram with number of genes present in each platform, genes in common between platforms, and genes in common among all three platforms.

Only genes common to all three platforms were used in the comparison. Genes were matched by UniGene Cluster (UniGene Build #161), and unique cluster memberships were determined for each array type, as listed in Table 1 and enumerated in the Venn diagram in Figure 1. The intersection for the three platforms consisted of 6430 UniGene clusters, and all analyses were carried out with all of these genes or a subset of them.

**Table 1:** Overlapping gene sets represented in 3 microarray platforms

	**Incyte UniGEM2**	**Hs-Operon V2**	**HG-U133A**
**Total features / probe sets:**	**9128**	**21522**	**22215**
**Distinct UniGene clusters:**	**8097**	**19179**	**13899**
	UniGem2 & Operon V2	UniGEM2 & HG-U133A	Operon V2 & HG-U133A
Genes in common	7082	6593	12999
Genes in common in all arrays	6430	6430	6430

### Comparison of expression ratios

An estimate of the concordance of the platforms was provided by the percentage of genes that showed expression ratios "in the same direction." All matched gene sets that had expression ratios (relative to the Universal Standard) greater than 1.0, irrespective of magnitude, were considered to be in the same direction, and the same for expression ratios less than 1.0. As seen in Table 2, the agreement was reasonably good, ranging from 74% to 82%. For comparison, ~50% concordance would be expected by chance. Many of the discordant values were at what might be considered "trivial" levels because a large proportion of ratios are near unity. For example, approximately 48%, 28% and 13% of the genes from Incyte, Operon and Affymetrix respectively, exhibit expression ratios within the range of 1.1 to 0.9. Thus, a ratio difference of only 0.95 versus 1.05 is designated a mismatch, which normally would be considered very concordant in the biological sense.

**Table 2:** Proportion of genes expressed in same direction between platforms ignoring expression level. The numbers of genes in matched and mismatched directions are given for each cell line and each platform pair, and agreement is given in percentages.

**Array Platform:**	**Cell line:**	**Direction-matched**	**Direction-mismatched (ignoring expression level)**	**Total number of genes used in calculation**	**Percent agreement in direction**
GEM2 & Operon V2	Jurkat	4597	1373	5970	77 %
	L428	3535	1251	4786	74 %
	SUDHL	4608	1525	6133	75 %
	Ocily3	4094	1368	5462	75 %
	LNCaP	4189	1333	5522	76 %
	MCF10A	4469	1429	5898	76 %
	Total	25492	8279	33771	75 %

GEM2 & HG-U133A	Jurkat	3657	906	4563	80 %
	L428	3203	918	4121	78 %
	SUDHL	3541	809	4350	81 %
	Ocily3	3826	984	4810	80%
	LNCaP	3747	1031	4778	78 %
	MCF10A	3584	950	4534	79 %
	Total	21558	5598	27156	79 %

Operon V2 & HG-U133A	Jurkat	3688	795	4483	82 %
	L428	2909	738	3647	80 %
	SUDHL	3520	776	4296	82 %
	Ocily3	3509	947	4456	79 %
	LNCaP	3607	860	4467	81 %
	MCF10A	3511	898	4409	80 %
	Total	20744	5014	25758	81 %

Statistical tests and thresholds on fold change are commonly used for the identification of altered gene expression by microarray assays. Hence, we estimated the statistical significance of differential expression of each RNA from the HUR by one sample Student-t test for a change from ratio of 1. Comparison between platforms using expression values significant at p < 0.05 shows concordance in direction of 93%–98% (Table 3 – no threshold). At the 1.5 and 2-fold threshold level, the agreement between platforms approaches 100% indicating that for reproducible data all three platforms are highly concordant in direction.

**Table 3:** Concordance between platforms using statistically significant expression ratios at p-value < 0.05 and at 1.5 and 2-fold threshold levels.^1^

	**No threshold**			**1.5-fold**			**2-fold**		
	**GEM2 vs. Operon**	**GEM2 vs. U133A**	**Operon vs. U133A**	**GEM2 vs. Operon**	**GEM2 vs. U133A**	**Operon vs. U133A**	**GEM2 vs. Operon**	**GEM2 vs. U133A**	**Operon vs. U133A**

**Number of comparisons^2^**									
Jurkat	1471	1678	1740	1096	1088	1220	588	614	749
L428	651	1394	750	580	1051	632	430	678	469
SUDHL	1400	1527	1707	1081	1177	1277	698	741	831
OCI-Ly3	1484	1834	1698	1285	1428	1333	814	900	903
LNCaP	1167	1170	2062	737	751	1252	369	404	696
MCF10A	1555	1429	1880	899	812	1118	405	409	559

**Percent of concordance**									
Jurkat	97%	96%	98%	98%	99%	100%	99%	99%	100%
L428	96%	96%	98%	98%	98%	99%	99%	99%	100%
SUDHL	95%	97%	98%	97%	98%	99%	99%	99%	100%
OCI-Ly3	94%	96%	96%	95%	98%	98%	98%	99%	99%
LNCaP	93%	95%	96%	97%	98%	99%	99%	100%	100%
MCF10A	94%	95%	96%	98%	98%	99%	99%	99%	100%

**Total number of comparisons**									
	7728	9032	9837	5678	6307	6832	3304	3746	4207
**Total number of concordances**									
	7334	8650	9540	5509	6191	6755	3257	3719	4190
**Percent agreement**									
	94.9%	95.8%	97.0%	97.0%	98.2%	98.9%	98.6%	99.3%	99.6%
**Percent of data used^3^**									
	54.0%	57.5%	60.4%	39.7%	40.2%	42.0%	23.1%	23.9%	25.8%

^1^GEM2: Incyte GEM2 arrays; Operon: Operon V2 arrays; U133A: Affymetrix Human Genome U 133A GeneChip arrays.

^2^Comparisons between two platforms were done only when the expressions of both platforms met the p-value and threshold criteria.

^3^Fraction of common expressions between two platforms to the total number of expressions found at p < 0.05 in both platforms.

### Correlation between platforms

**Figure 2:**
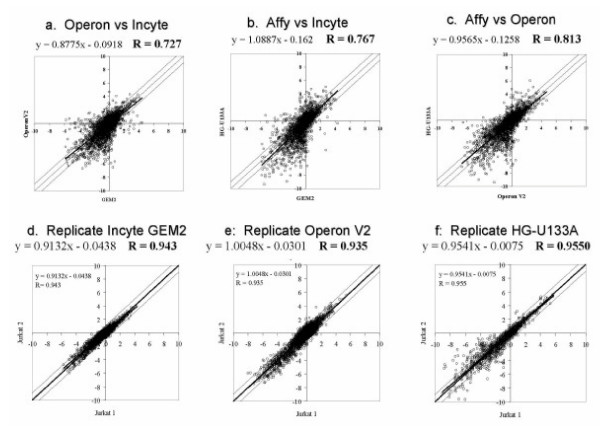
**a-f**. Scatter plot analysis to determine correlation coefficients between and within platforms using Jurkat RNA as an example. Correlations for all cell lines are given in Table 4. (a) Operon versus Incyte (b) Affymetrix versus Incyte (c) Affymetrix versus Operon (d) GEM2 versus GEM2 replicate correlation (e) Operon versus Operon (f) HG-U133A versus HG-U133A

The scatter plots shown in Figures 2a–c indicate graphically the correlation between the platforms for Jurkat RNA. Values of r for all platforms and all cell lines are given in Table 4. The values ranged from 0.7 to 0.8. Operon and Affymetrix were better correlated than were Operon and Incyte. The values were intermediate for the relationship between Incyte and Affymetrix. Figures 2d–f and Table 5 indicate that the correlation coefficients between replicates within the same platform are closer, as expected, when compared to the correlations between platforms. In general, however, there was reasonably good concordance among the platforms.

**Table 4:** Cross platform correlation coefficients. Pearson correlation coefficients are given for each platform pair and cell line, calculated over the genes common to all platforms. Values were obtained from scatter plots as shown in Figure 2a-c.

**Platform**	**Jurkat**	**L428**	**SUDHL**	**OCI-Ly3**	**LNCaP**	**MCF10A**	**Median**
Incyte/Operon	0.727	0.707	0.708	0.724	0.710	0.708	0.709
Incyte/Affy	0.767	0.777	0.781	0.780	0.741	0.744	0.772
Operon/Affy	0.813	0.784	0.783	0.790	0.796	0.782	0.787

**Table 5:** Median correlation coefficients of replicates within same platform. Calculations and values obtained as in Table 4.

**Platform**	**Jurkat**	**L428**	**SUDHL**	**OCI-Ly3**	**LNCaP**	**MCF10A**
GEM2	0.859	0.919	0.902	0.873	0.827	0.845
Operon V2	0.916	0.805	0.849	0.828	0.951	0.928
HG-U133A	0.956	0.957	0.960	0.952	0.963	0.954

### Principal Component Analysis (PCA)

**Figure 3:**
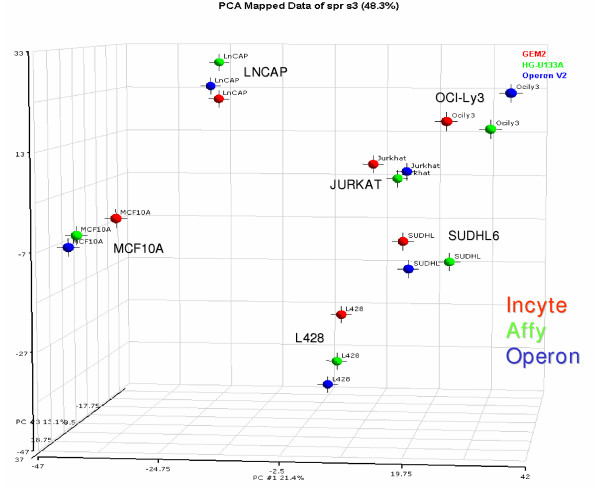
Principal Component Analysis (PCA) of the three microarray platforms and six cell lines using expression of the 3186 genes with signals above background.

A projection on the first three principal components, which together explain 48.8 % (21%, 14%, and 13%) of the total variance, is shown in Figure 3. Close clustering of the cell samples is observed in this projection, indicating appreciable agreement among array platforms.

### "Correlation of Correlations" analysis

**Figure 4:**
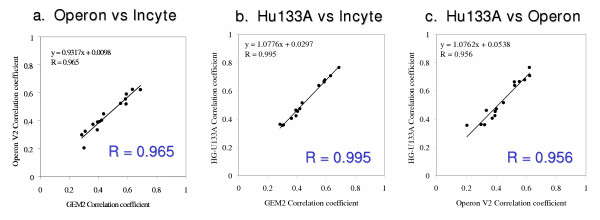
**a-c**. Correlation of correlations of platforms for all cell lines. Correlation values R for each pair of platforms are given in the figures. (a) Operon versus Incyte (b) Affymetrix versus Incyte (c) Affymetrix versus Operon.

The global concordance of the three platforms across all of the cell lines was estimated by calculating the "correlation of correlations" coefficient [10,11]. As seen in Figure 4a–c, the correlations for the three platforms across all cells lines were quite good. The Pearson correlation of correlation coefficients was 0.965 between Operon and Incyte, 0.995 between Affymetrix and Incyte, and 0.956 between Operon and Affymetrix.

### Clustered Image Map (CIM) visualization and analysis [12]

**Figure 5:**
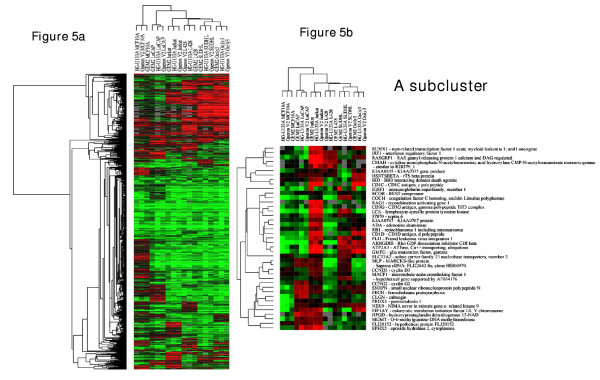
**a-b**. Clustered image maps showing patterns of expression relationship among genes, platforms, and cell lines. The axes were ordered by hierarchical clustering using an uncentered correlation and the average linkage algorithm for 909 genes expressed at a two-fold or greater level in at least two of the six cell lines. (a) Clustering of all 909 genes (b) A subcluster of 41 genes to show correct clustering and congruence of expression values. As indicated by the cluster trees, all three platforms gave essentially the same relationships among the six cell lines.

We used hierarchical clustering to demonstrate graphically the relationships among platforms, among cell lines, and among genes. 909 genes expressed at two times background or more in at least two of the six cell lines were included in the analysis. The resulting CIM ("heat map") is shown in Figure 5a–b. All three platforms cluster together for all six cell lines, as one would wish to find, and almost all of the gene expression values show reasonable concordance. Only a few exceptions can be seen in the cluster shown in Figure 5b. Some of the mismatches may be due to simple errors in gene identification, rather than to the technologies of the platforms. The Incyte library is guaranteed by the manufacturer to be only 90% correct, and an unknown percentage of the Operon and Affymetrix oligonucleotides may have been designed on the basis of incorrect sequences in the public databases. Indeed, we found one oligonucleotide in the Operon set that was apparently designed from an EST sequence that has since been withdrawn from the UniGene database (see RT-PCR studies below). In any case, the concordance is quite high across all platforms with this method of analysis as well as with the others.

### Quantitative real-time RT-PCR

**Figure 6:**
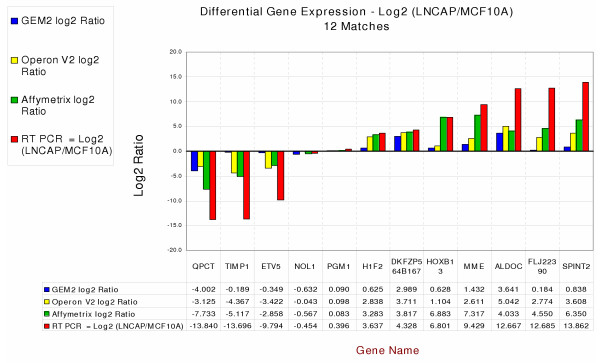
Quantitative real-time RT-PCR analysis of 12 genes matched for direction of expression relative to the reference RNA for all three platforms. Log2 ratios are given in table below the graph. This example is a comparison between LnCaP and MCF-10A.

In a pilot study with the three platforms, we compared and contrasted gene expression values for only the cell lines MCF10A and LNCaP. RT-PCR data for twelve genes are shown in Figure 6. Most of the values are in reasonable agreement except that there are differences in the *magnitudes *of the expression ratios. As found in other studies, the RT-PCR values are generally higher, probably because ratios are "flattened" with the microarray platforms. Affymetrix ratios are sometimes higher, but that may simply reflect the method of quantitation used in their analysis. The cDNA array ratios are generally lower than those from other platforms. Because the cDNA fragments are longer and double-stranded, they are more prone to non-specific hybridization and can cross-hybridize more easily to related sequences. These characteristics of the probes may result in higher background signal and concomitant reduction in dynamic range of the ratios. In general, we have found that the long oligonucleotide arrays have a larger dynamic range than the do the cDNA arrays.

**Figure 7:**
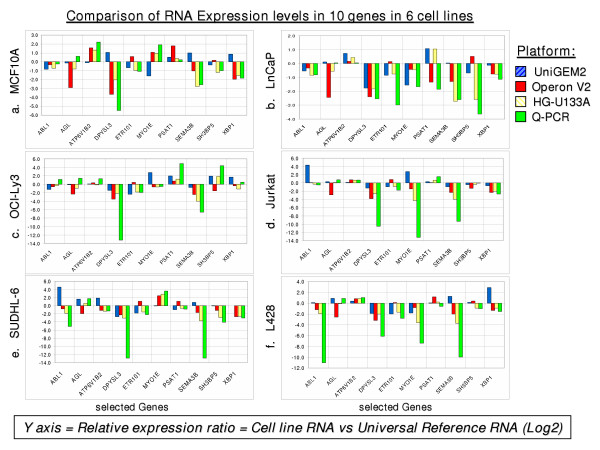
**a-f**. Quantitative RT-PCR analysis of 10 mismatched genes in the six cells lines for all three platforms. (a) MCF10A, (b) LnCaP, (c) OCI-Ly3, (d) Jurkat, (e) SUDHL-6 and (f) L428.

For further RT-PCR analysis, we chose a set of ten genes to test the accuracy of the three array platforms for all six cell lines. Those genes were chosen because there appeared to be a discrepancy among platforms in the direction of their ratios (i.e., whether they ratios were greater or less than unity). The results are shown in Figures 7a–f. Of special interest was gene ETR101, in which the Operon array was in disagreement across all cell lines. Further inquiry revealed that the sequence had been found to be incorrect and had been removed from the UniGene database. Since the oligonucleotide had been designed from the incorrect sequence, it is not surprising that it gave a different value. Other discrepancies may be due to similar sequence errors, as even the most up-to-date databases are still being corrected and modified. In the case of AGL, the RT-PCR assay is in disagreement in several cases with two out of three of the array platforms; it appears to demonstrate an upregulation of the mRNA, whereas the arrays, with the exception of Incyte, point to a downregulation. Although RT-PCR is supposed to be the "gold standard" for measuring gene expression, this result shows that caution is indicated in interpreting results with even the PCR technology.

## Discussion

The purpose of this study was to compare and contrast the three major microarray platforms, with the goal of qualifying the long oligonucleotide platform for general use in our laboratories. Our principal findings were that the *magnitude *of any particular expression ratio may differ from one platform to the other but that the "direction" of gene expression difference for genes with sufficient intensity of signal appears to be well correlated across platforms. The differences in apparent magnitude of expression were not unexpected. The platforms differ widely in many characteristics, including size of targets, number of targets, concentration of targets, labeling protocol, and quantitation algorithms. Nevertheless, the overall concordance of the three platforms was reasonably good, and one should be able to compare experimental data between and among the different array types. That is perhaps not surprising if one considers microarrays simply as miniaturized, multiplexed dot blots.

A survey of the literature on platform comparisons reveals a mix of results. Several studies suggest disagreement in expression between platforms [13-20], and several demonstrate agreement [11,21-34]. The explanation for the discrepancies among these reports is not altogether clear. However, we think the following points should be remembered when designing such studies:

### The same RNA templates should be used throughout the entire experiment

RNA preparations from similar, but non-identical, biopsy samples can yield dissimilar results [35], and RNA from different versions of the same cell line can yield widely different expression profiles [36,37].

### Only genes common to all platforms should be used in the analysis

That may seem an obvious requirement, but it is not always easy to meet in practice. For example, careful study of sequences present in various cDNA arrays indicates that 20–30% of spots may contain the wrong clone or a misidentified one [10,14,38-40]. A similar situation exists for the short oligonucleotide platform (Affymetrix) because more than 19% of the sequences have been reported not to correspond to the appropriate mRNA Reference Sequence [41,42]. In these studies, measurements of cross-platform consistency were significantly improved when only sequence-matched genes were used. Similarly, discrepant results in studies using cDNA and Affymetrix platforms could be explained by errors in gene sequences [43]. The percentage of incorrect or misidentified sequences in the long oligonucleotide libraries is unknown, but we appear to have found one sequence in error during our own RT-PCR studies. Overall, then, significant disagreement in expression ratios among platforms may be due to sequence errors, not to intrinsic differences among array systems.

### Only spots with signals significantly above background (and that pass stringent quality-control filters) should be used in the comparisons

With most high-density arrays, a large proportion of the genes can be expected to have signals near background, as probably fewer than half of all human transcripts are expressed in any particular cell type or tissue [44]. Although stringent filtering decreases the number of measurements included in the analysis, the outcome will be more meaningful.

When comparing results from the three platforms, the magnitudes of ratios were often quite different, but there was generally good agreement in the direction of differences in expression (Figs. 6 &7, Tables 2 &3). Thus, it may be less productive to correlate absolute changes in expression than it is to look for agreement in direction.

Overall, the two oligonucleotide platforms were the most concordant pair. Possible reasons are (i) that the array targets are single stranded, all of the same size (25-mer or 70-mer), with approximately the same melting temperature; (ii) the array elements have a much higher molarity of gene-specific sequences than do the cDNA arrays; (iii) that oligonucleotides tend to be more specific in terms of sequence and less prone to cross-hybridization than are cDNAs.

## Conclusion

As the efficiency and economy of oligonucleotide arrays improves, they will probably become the platforms of choice for gene expression analysis, replacing the cDNA type entirely. Long-oligonucleotide arrays are being produced by ink-jet [6,45] and pin-spotting methods [26,27,31]. In these studies, where experiments specifically compared long-oligonucleotide arrays with the more "standard" platforms, correlations were good, and the long-oligonucleotide arrays performed as well as, or better than, the cDNA or short oligonucleotide variety. The data reported here confirm and extend those results, with the added advantage of comparing all three major platforms at one time and in the same place – something that, to our knowledge, has not been done before. As a note of caution, however, we have used materials from three commercial array platforms, and our results and conclusions may not necessarily translate to other platforms and manufacturers.

Since this study began, many articles have appeared reporting the "meta-analysis" of microarray data from unrelated laboratories using several different array platforms [46-54]. Those studies have reported useful clinical and diagnostic findings when the data were stringently filtered before analysis. Eventually, standardization and simplification of the systems may lead to a situation in which only one or two robust methods are used in all laboratories, with a concomitant improvement in the accuracy of gene expression data.

## Methods

### Experimental design

Three array platforms were tested. Incyte cDNA arrays and Operon long oligonucleotide (70-mer) arrays were printed in house, and Affymetrix 25-mer HG U133A arrays were purchased from the manufacturer. The cDNA and long oligonucleotide arrays were assayed in quadruplicate, two each of red/green and green/red for dye "flips" (reverse fluor experiments) to help eliminate dye bias (except for L428 that had 3 replicates and OCI-Ly3 with 5 replicates). The Affymetrix arrays were tested in duplicate. RNA preparations from six cell lines were tested with each platform using a universal reference RNA (Stratagene) as the standard.

### Cell lines and RNAs

Cell lines were grown and RNA isolated at the core Gene Expression Laboratory, NCI-Frederick. MCF10A (benign mammary epithelial), LNCaP (prostate carcinoma), Jurkat (T-cell lymphoma), SUDHL6 (germinal center B-cell like diffuse large B-cell lymphoma), OCI-Ly3 (activated B-cell like diffuse rare B-cell lymphoma and L428 (Hodgkin's lymphoma) were grown under standard conditions [55], and RNA was isolated from the cells using TriReagent following the manufacturer's protocol (Molecular Research Center, Inc., Cincinnati, OH). Integrity of the RNA was confirmed by analysis with the Agilent 2100 Bioanalyzer (Palo Alto, CA) using the RNA 6000 LabChip^® ^kit. For use as the index standard, Human Universal Reference RNA (HUR RNA) was purchased from Stratagene (La Jolla, CA).

### Preparation of cDNA and oligonucleotide arrays

Arrays with 10,000 cDNAs were prepared from ready-to-print UniGEM2 libraries obtained from Incyte, Inc. (Wilmington, DE). Version 2.0 libraries containing 22,000 oligonucleotides of 70 bases in length were obtained from Operon, Inc. (Alameda, CA). Arrays were printed by standard protocols on Corning Ultra-GAPS II slides (Corning, NY) using a GeneMachine^® ^(San Carlos, CA) instrument. cDNAs were suspended at a concentration of 100 μg/ml and oligonucleotides at 25 μM in 3XSSC buffer, and the arrays were printed using SMP3 pins from Telechem International (Sunnyvale, CA). The spotted nucleic acids were fixed to the slides using protocols supplied by the manufacturers. The 25-mer oligonucleotide HG U133A Genechip^® ^arrays were purchased from Affymetrix, Inc (Santa Clara, CA).

### Labeling and purification of targets

Labeled cDNAs from cell samples for hybridization to the long-oligonucleotide and cDNA arrays were synthesized and labeled by the indirect amino-allyl method, using reagents and protocols supplied by the manufacturer. For cDNA synthesis, we used Stratascript reagents (Stratagene, La Jolla, CA) and Cy3 and Cy5 fluorophore amino-allyl reagents from Amersham (Piscataway, NJ). Twenty micrograms of total RNA was used for each synthesis. Labeled cDNA targets were purified using Minelute purification kits (Qiagen, Valencia, CA). cRNA targets for the Affymetrix arrays were synthesized, labeled, and purified according to vendor's (Affymetrix) instructions. Briefly, 10 μg of total RNA was used to make double-stranded cDNA using reagents and protocols obtained from Invitrogen (Carlsbad, CA). Linear amplification was carried out by a modification of the Eberwine T7 method [56], and biotin was incorporated into the cRNA using the Enzo High Yield RNA Transcript labeling Kit (EnzoDiagnostics, Farmingdale, NY).

### Hybridization and washing of arrays

The cDNA and long-oligonucleotide microarrays were prehybridized in 40 μl of 5XSSC with 0.1% SDS and 1% BSA at 42°C for 30 minutes. The prehybridization solution was removed, and arrays were hybridized for 16 hours at 42°C in 5XSSC buffer containing Cy3/Cy5 labeled targets, 25% formamide, 0.1% SDS, 1 μg Cot-1 DNA, and 1 μg poly A RNA. The cDNA arrays were washed at room temperature in 2XSSC, 0.1% SDS for 2 minutes, 1XSSC for 2 minutes, 0.2XSSC for 2 minutes and 0.05XSSC for 1 minute. The long-oligonucleotide arrays were treated the same except that the last wash step was omitted. The slides were dried by centrifugation at 650 rpm for 3 minutes. The Affymetrix arrays were hybridized and washed using the manufacturer's protocol. The arrays were then stained with streptavidin-phycoerythrin using the standard antibody amplification protocol (GeneChip^® ^Expression Manual, Affymetrix, Inc., Santa Clara, CA).

### Array scanning and image processing

Long-oligonucleotide and cDNA arrays were scanned using an Axon 4000B scanner at 10-micron resolution. Images were processed, and signals from spotted arrays were quantitated using Genepix 3.0 software (Axon Instruments, Union City, CA). The Genepix result files, including signal, background, standard deviation, pixel statistics and quality parameters for both channels were deposited in the microarray database (mAdb) maintained by the NCI/CIT bioinformatics group [57]. The data were filtered on the basis of signal levels and spot quality. Local background values were subtracted from spot intensities to obtain signal values. Data were included if the signal-to-background ratio was ≥ 2, the signal intensity was >100, the spot diameter was between 50 and 180 microns, at least 70% of the pixels were above their standard deviation and the spot was not flagged as "bad" visually. Arrays were normalized by median-centering the logarithmic ratios so that the median ratio of all genes that passed through the filters was equal to 1. For cDNA arrays, normalized expression ratios of 9050 genes were calculated, and the same procedure was applied to long-oligonucleotide arrays for the expression of 20,799 genes. Affymetrix HG-U133A arrays were scanned with the Affymetrix GeneArray scanner at 488 nm and 3-micron resolution. The images were analyzed using Microarray Suite 5.0 software (MAS5; Affymetrix Inc., Santa Clara, CA). Cell-line to HUR expression ratios were computed by comparative analysis of MAS5 values. The data were filtered using MAS5's signal detection and change calls generated at recommended default settings. The ratios included were those that had present calls for signal detection or an increase or decrease call associated with the ratio. The filtered data contained 17,647 genes. For all statistical calculations, logarithmic values of ratios to the base 2 were used.

### Determination of genes in common among all platforms

Genes were matched by UniGene cluster methods [11,58], and expression levels were compared for only the 6,430 genes common to all platforms (Table 1 and Figure 1). UniGene clusters (Homo sapiens: UniGene Build #161) of probes of all three platforms were determined by the NCI/CIT Bioinformatics group [57] using the BLAST program from the National Center for Biotechnology (NCBI, Bethesda, MD 20894). There were multiple probes for some of the UniGene clusters. This resulted in matching of two or more probes of one platform to one or more probes of another platform. All possible combinations across three platforms were considered for each UniGene cluster. One probe from each of the platforms was selected as follows. Initially, for a given combination, the replicates were averaged to obtain expression patterns of six RNAs on three platforms. The sum correlation coefficients of these three patterns to their mean pattern was determined. The combination having the highest sum was selected for further analysis. This method relies on the assumption that the probes specific for a gene yield similar expression patterns independent of the platform. The matched expression ratios will be made available at our website .

### Estimation of total matched versus mismatched expression values

Expression ratios for genes in common across all cell lines and platforms were determined. If the ratio of a gene was ≥ 1 or ≤ 1 for both platforms being compared, the expression was considered matched irrespective of the magnitude. Otherwise, the ratios were considered to be mismatched (i.e., in opposite directions). These values give a rough, binary indication of the correlation between platforms (Table 2). Concordance between platforms using significant expression ratios at p-value <0.05 and 1.5 to 2-fold threshold levels are given in Table 3.

### Correlation of expression among all platforms

To determine how well the data from the three platforms coincided, correlation coefficients were obtained from analysis of scatter plots of the mean expression values from the three array formats. Figure 2a–c are examples of scatter plot analyses of the three platforms using data from the Jurkat cell line RNA. The boundaries for ratio values greater than two-fold are delineated by the external lines. Correlation coefficients for all three pairs of platforms for all six cell lines are listed in Tables 4 &5).

### Principal Component Analysis (PCA)

The global gene expression patterns of the six cell lines in all three platforms were studied by principal component analysis (PCA) [59]. All genes (3186) with signals above background were included. Differences in signal magnitude among platforms were nulled out by normalizing the data from each to unit standard deviation. Individual platform variations were accounted for by employing unit variance. A projection on the first three principal components, which explained 48.8% (21%, 14%, and 13%) of the total variance, is shown in Fig. 3. The calculations were done using Partekpro 5.0 software (Partek Inc., St. Charles, MO)

### Platform concordance by "correlation of correlations" coefficient

The global concordance of expression levels of the three platforms can be expressed in terms of the 'correlations of correlations' coefficient described previously [10,11]. To perform the computations, step 1 was calculation of the Pearson correlation coefficient across all matched genes for each of the 15 possible pairs of cell lines for each platform. Step 2 was calculation of the Pearson correlation coefficients of those correlation coefficients for the three possible ways of pairing the three platforms. The results are shown in Figures 4a–c.

### Hierarchical cluster analysis and Clustered Image Maps (CIMs)

Hierarchical clustering of individual replicates (data not shown), including 3662 genes detected in 80% of the arrays revealed a grouping of RNA samples independent of the type of array platform. A set of 909 genes expressed at >two-fold levels in all platforms in at least two cell lines was used for hierarchical cluster analysis to determine how closely the genes, cell lines and platforms corresponded in their expression values. As a distance metric, we used 1-r, where r is the Pearson correlation coefficient [60]. Cluster nodes were determined using an average linkage algorithm. In the resulting CIM (heat map) [14a], up- and down-regulation with respect to the reference RNA are color-coded as red and green, respectively (Figure 5a–b).

### Quantitative real-time RT-PCR

RNA preparations from each of the six cell lines and Stratagene Human Universal Reference RNA were converted into single-stranded cDNA using the Applied Biosystems High-Capacity cDNA Archive kit (ABI, Foster City, CA). Primer and probe sets were obtained as ABI Assays-on-Demand™ Gene Expression Products (TaqMAN^® ^MGB probes, FAM™ dye-labeled) for a set of genes to be studied, as well as GAPDH and BACT, which were used as comparative controls. All quantitative PCR reactions were performed in quadruplicate, with two carried out in an ABI Prism^® ^7000 sequence detection system and two in a Corbett Research R-300 instrument (Corbett Research, Sidney, Australia). The results were analyzed using the "Relative Quantitation of Gene Expression" method described in ABI Prism 7700 Sequence Detection System User Bulletin #2, Rev B. An initial study comparing only two cell lines, LnCaP and MCF10A, was carried out for the three platforms. Twelve genes that matched in direction of change were chosen for Q-PCR analysis, and the results are shown in Figure 6. Subsequently, ten genes were chosen for analysis using all six cell lines and three platforms. Genes in that set were deliberately chosen for mismatched ratios to determine if any platform was in error more often than the others. The results are shown in Figures 7a–f.

## Abbreviations

HUR RNA: Human Universal Reference RNA

mAdb: microarray data base

## Competing interests

The author(s) declare that they have no competing interests.

## Authors' contributions

DP, JG, JH, JP, JH, and RP worked on developing the long oligonucleotide microarray system and provided the raw data and initial array analysis for both the cDNA and long oligonucleotide arrays. CHK, DM and LG provided input for the long oligonucleotide development and provided the Affymetrix chip data and analysis. GVRC provided statistical analysis of microarray data for the cross platform comparisons of this study. ESK drafted the original manuscript and JW was the main critical reviewer of the manuscript. LS, JCB and JeG provided valuable input into the design of the original experiments and were crucial in support of the new array development. All authors read and approved the manuscript.
